# Specific-Locus Amplified Fragment Sequencing Reveals Spontaneous Single-Nucleotide Mutations in Rice* OsMsh6* Mutants

**DOI:** 10.1155/2017/4816973

**Published:** 2017-05-15

**Authors:** Hairui Cui, Qiongyu Wu, Bin Zhu

**Affiliations:** Institute of Nuclear-Agricultural Sciences/Key Laboratory of Chinese Ministry of Agriculture for Nuclear-Agricultural Sciences, Zhejiang University, Hangzhou 310029, China

## Abstract

Genomic stability depends in part on an efficient DNA lesion recognition and correction by the DNA mismatch repair (MMR) system. We investigated mutations arising spontaneously in rice* OsMsh6* mutants by specific-locus amplified fragment sequencing. Totally 994 single-nucleotide mutations were identified in three mutants and on average the mutation density is about 1/136.72 Kb per mutant line. These mutations were relatively randomly distributed in genome and might be accumulated in generation-dependent manner. All possible base transitions and base transversions could be seen and the ratio of transitions to transversions was about 3.12. We also observed the nearest-neighbor bias around the mutated base. Our data suggests that* OsMsh6 (LOC_Os09g24220)* is important in ensuring genome stability by recognizing mismatches that arise spontaneously and provides useful information for investigating the function of the* OsMsh6* gene in DNA repair and exploiting MMR mutants in rice induced mutation breeding.

## 1. Introduction

The genome of a living organism is continuously subjected to a wide variety of genotoxic stresses from endogenous or exogenous DNA damaging agents during its lifecycle. Several DNA damage repair systems have been formed in the process of evolution, to sense, recognize, and eliminate the incurred damage to the genome [1–3]. The mismatch repair (MMR) is a major DNA repair pathway whose function is critical for maintaining genome stability and DNA replication fidelity by recognizing and repairing erroneous insertions, deletions, and misincorporation of bases during DNA replication, genetic recombination, and repair of some forms of DNA damage [4–7]. The major components in the MMR system include MutS, MutH, and MutL in* Escherichia coli* [8]. MutS forms a homodimer that recognizes and binds a mismatched base. MutL homodimer binds the MutS-DNA complex and acts as a mediator between MutS2 and MutH, resulting in MutH activation. The activated MutH nicks the unmethylated strand at the GATC site. Subsequently, the error-containing segment is removed by exonuclease and the gap is filled by a new strand synthesized by DNA polymerase III and DNA ligase [5, 7]. In eukaryotes, multiple homologues of MutS (MSH1–MSH7) and MutL (MLH1–3, PMS1, and PMS2) have been characterized, but not of MutH. Among them, MSH2 and MLH1 are the key monomers that form heterodimers with other MMR proteins, such as MSH2–MSH6 (MutS*α*), MSH2-MSH3 (MutS*β*), MSH2–MSH7 (MutS*γ*), MLH1-PMS1 (MutL*α*, for humans, MLH1-PMS2), and MLH1–MLH3 (MutL*γ*) [9–13]. MutS*α* is mainly required to correct single base mispairs and short insertion/deletion loops, whereas MutS*β* is predominantly involved in the removal of large insertion/deletion loops (2–12 nucleotides), and plant specific MutS*γ* preferentially recognizes certain base-base mismatches [11, 14]. Thus mutation or disruption of plant* MutS* and* MutL* genes may affect DNA mismatch repair, resulting in mutations in both morphology and DNA. MMR mutants showed an expected increased frequency of point mutations and genome instability in* A*.* thaliana* [15–18], rice [19], and tobacco [20]. Loss of the MMR activity in plants specifically affected morphology, fertility, and seed development in a generation-dependent manner [17, 21, 22].

Rice is an important food crop and a prominent molecular model species for monocotyledonous plants. Some MMR genes have been annotated in Rice Annotation Project Database. Based on comparative genomics, 12 MMR genes have been identified in rice using similarity searches and conserved domain analysis [23], one of which is* OsMsh6 (LOC_Os09g24220)*, a homologue to* AtMsh6 (At4g02070)* in the MMR system of* A. thaliana*. However, the biological function of* OsMsh6* awaits further investigation. In this study, we investigated the single-nucleotide mutations of rice* OsMsh6* mutants by specific-locus amplified fragment sequencing (SLAF-seq) [24]. Our data indicates that frequency of single-nucleotide mutations is dramatically increased in* OsMsh6* mutants and suggests that* OsMsh6* is important in ensuring genome stability by recognizing mismatches arising spontaneously.

## 2. Materials and Methods

### 2.1. Plant Materials

By a BLAST search against flanking sequences in Rice Tos17 Insertion Mutant Database (http://tos.nias.affrc.go.jp), mutant seeds of* LOC_Os09g24220* were introduced from National Institute of Agrobiological Sciences, Japan. Three homozygous insertion mutants of* OsMsh6 (LOC_Os09g24220)* derived from Nipponbare were obtained at T3 generation after molecular analyses, and the* Tos17* insertion position is at 1st exon, 8th exon, and 3′-UTR in NF9010, NF7784, and ND6011, respectively [25, 26]. The wild type, Msh6WT without* Tos17* insertion from the segregating generation, was also harvested and used as the control to eliminate the mutations caused by the somaclonal variation in mutants during the tissue culture.

### 2.2. Genomic DNA Extraction

The leaf tissues were sampled from Msh6WT and four independent lines for each mutant in different generations, termed as NF9010/G4 to NF9010/G7, NF7784/G4 to NF7784/G7, and ND6011/G4 to ND6011/G7, respectively. Genomic DNA was extracted according to the method described by Murray and Thompson [27]. DNA quality and concentration were evaluated using a NanoDrop ND-1000 spectrophotometer and 1.5% agarose gel electrophoresis.

### 2.3. SLAF-Seq

SLAF-Seq was conducted according to the method described by Sun et al. [24].

**Figure 1:**
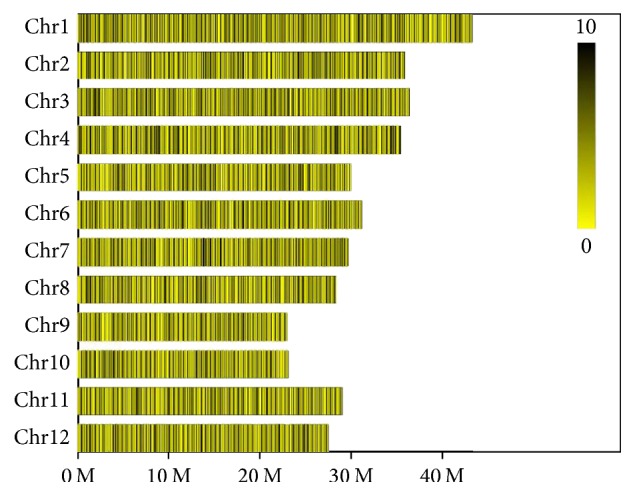
Distribution of SLAF tags in genome. Each yellow row represents a chromosome. The color scale from yellow to black represents the number of SLAF tags (0 to 10): the deeper color, the higher number of SLAF tags.

Genomic DNA was digested with the restriction enzyme HaeIII. The obtained fragment (SLAF tag) was processed to add A to 3′ end and connected to dual-index adapter [28]. After PCR amplification and purification, fragments mainly with 394–414 bp in size were isolated and then subjected to PCR amplification for sequencing by Illumina HiSeqTM2500. Main parameters related to SLAF tags developed and sequence data in this study were listed in Figure 1 and Table 1.

### 2.4. SLAF-Seq Data Grouping and Sequence Comparison

The SLAFs were identified and filtered to ensure that the original sequencing data were effectively obtained. All SLAF pair-end reads with clear index information were clustered based on sequence similarity using BLAT [29]. The sequences with good quality from mutants at four generations and Msh6WT were compared to check the base variation. When a base from the sequence of mutants is different with Msh6WT, it is considered as a mutated base.

## 3. Results

### 3.1. Distribution of Mutated Bases and Mutation Density

**Figure 2:**
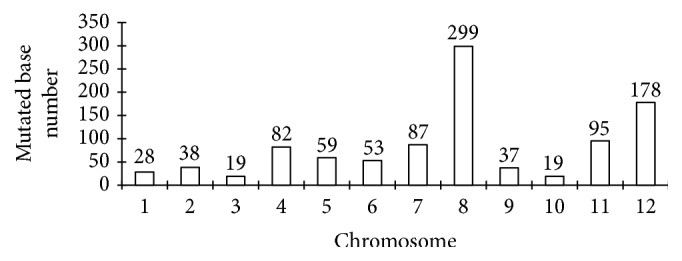
Number of mutated bases on different chromosomes.

After stringent filtering, polymorphic SLAF sequences were extracted and compared between mutant lines and Msh6WT. A total of 994 mutated bases were retained (Supplementary File in Supplementary Material available online at https://doi.org/10.1155/2017/4816973). This result suggests that spontaneous single-nucleotide mutations occur in* OsMsh6* mutants. Among these mutations, 470 were found only in either of three mutants, whereas 278 and 46 were observed simultaneously in two and three mutants, respectively. These spontaneous mutations were found on all chromosomes and distributed as relatively random as SLAF tags in whole genome, but the mutation number was uneven on different chromosomes. The highest was found on chromosome 8 (299), followed by chromosome 12 (178), together accounting for nearly a half of all mutations, with less than 100 on each of another 10 chromosomes (Figure 2).

The number of mutated bases discovered by SLAF-Seq varied with mutants and reproductive proceeding generations (Table 2). Totally more mutated bases were found in the mutants NF7784 and NF9010 than that in the mutant ND6011, and same situation was present at three of four generations, except for G6 generation. This may be related with the* Tos17* insertion position which causes the disruption of* OsMsh6* function in different degree. When we checked the number of mutated bases in different mutants at different generations, we observed the same tendency: the more reproductive proceeding generations, the more mutations. It is suggested that mutations can accumulate as the reproductive generations proceed.

Taking the SLAF number (Table 1) and length described in the methods and the mutations detected, we calculated the density of single-nucleotide mutations for each mutant line (Table 2). It ranged from 1/8.42 kb to 1/307.63 kb, with an average about 1/136.72 Kb.

### 3.2. Mutation Spectrum

To explore the spectrum of spontaneous mutations in* OsMsh6* mutants, we analyzed the number and characteristics of four kinds of mutated nucleotides (Table 3). The number of mutated A or T was a little more than expected, but mutated C was significantly less. Transition and transversion mutations accounted for about 3/4 and 1/4 of mutations, respectively. G and C showed higher transition ration than A and T. On average, about 82% of mutations were heterozygous, which is higher than expected. We also observed that the mutated base at same position was heterozygous in one mutant but homozygous in another mutant; this kind of mutations accounts for about 9.4% of total mutations.

We also counted the number of different base substitutions (Table 4). All possible base transitions and transversions were found, indicating a wide mutation spectrum in* OsMsh6* mutants. Similar frequency was observed for A → G, C → T, and T → C transitions, but a little low for G → A transition. A *↔* C and C *↔* G were the highest and lowest transversion mutations, respectively, while A *↔* T and G *↔* T transversion showed similar intermediate frequency.

### 3.3. Local Compositional Biases of Mutated Bases

When we examined nucleotide positions flanking the mutated bases, we detected deviations from random expectations on both sides (Table 5). 10 of 24 flanking positions for all mutated bases were found with significantly higher base bias than expected, which included six upstream sites and four downstream sites (*P* < 0.05).

In general, frequency of purines (50.3%) flanking all mutations was almost equal to pyrimidines (49.7%), but the situation is different from upstream to downstream side. At upstream side the present frequency of purines (47%) was lower than that of pyrimidines (53%), while at downstream side the frequency (53.7%) of purines was higher than that (46.3%) of pyrimidines (Supplementary Table 2). Different mutations, even within mutated purines or pyrimidines, showed different base bias patterns. For example, when the mutated base was A, C at −1 and T at +2 and +3 were more frequent, while C at +3 and G upstream and at +2 were less frequent; when the mutated base was G, A at +2 and +3, C at −1 and −2 and T at −3 were more frequent, whereas C at +2 and +3 and G a upstream were less frequent. After comparison with the expected frequency, we found 15 bases at 38 positions (Table 6) with the deviation frequency at least 15% less or more than that of random expectation.

## 4. Discussion

It has been reported that MutS deficiency caused genome instability and increased mutation rates in* Arabidopsis* [16–18]. Suppression of MMR system through a dominant negative strategy also could produce high mutation rates in* Arabidopsis* [15] and rice [19]. In this study, we demonstrate that* OsMsh6* deficiency resulted in spontaneous generation of a wide variety of single-nucleotide mutations. Therefore the mutated* OsMsh6* can be regarded as a mutator which persisted during the life cycle of a plant and the stronger mutation effects can be expected compared with mutagen treatment. Indeed we detected 994 mutations in about 4.3 Mb sequences and on average the single-nucleotide mutation density is about 1/136.72 Kb for each mutant line. The result demonstrates that loss function of* MutS* is as efficient in producing point mutations in rice as that in* Arabidopsis*. As MMR is a conservative DNA repair pathway present in different organisms, our results suggest that the* OsMsh6* gene plays an important role in mismatch repair in rice. The higher heterozygous mutations observed in our experiments might be the newly arising and unrepaired ones.

Instead of causing DNA damage by chemical and radiation treatments, negative regulation of the DNA repair system is expected to have the same effects on mutant production. Thus, the DNA mismatch repair system (MMR) might be a good target for establishing induced mutation system. As a chemical mutagen, ethyl methane sulfonate (EMS) has been widely used for generating point mutations to enhance genetic diversity in plants. EMS treatment almost exclusively produced G:C to A:T base substitution in some specific genes of* Arabidopsis thaliana* and maize [30, 31]. In addition to the large number of mutations, we found all possible base transition and base transversion mutations in* OsMsh6* mutants. Our results indicate a wider spectrum of spontaneous mutations caused by disruption of* OsMsh6* gene in rice, and the mutation spectrum is different from that induced by EMS. Therefore, the manipulation of MMR repair process might produce different mutation types from those produced by mutagenic treatment.

Many varieties and a large number of mutants have been obtained through induced mutation approach, but the mutation in most of them is involved in single gene or few loci. In* C*.* elegans*, multigeneration propagation of parallel MSH2-deficient subcultures resulted in relatively rapid accumulation of microsatellite shifts and elevated reversion of a dominant point mutation [32]. In* Arabidopsis*, the fifth-generation lines of* Atmsh2-1* mutant rapidly accumulated microsatellite mutations and a wide variety of abnormalities in morphology and development, fertility, germination efficiency, seed development, and seed set [17]. Consistent with these two reports, we observed significant variations in several agronomic traits of* OsMsh6* mutants in our previous study [25, 26] and we also found that single-nucleotide mutations could be accumulated in generation-dependent manner in this study. The generation-to-generation accumulation of mutations caused by MMR-deficiency is helpful to obtain the mutant with multiple locus mutations or phenotypes. This may be important when alteration of a multiple locus trait is desired, because mutagen treatment sufficient to introduce the necessary multiple mutations might bring unacceptable damage to organism. The combination of these two strategies may be great potential to obtain higher mutation frequency and to improve the effectiveness of induced mutation breeding.

## 5. Conclusion

We analyzed the spontaneous single-nucleotide mutations in rice* OsMsh6* mutants. Results suggest that* OsMsh6* is important in ensuring genome stability by recognizing mismatches that arise spontaneously. Our data provides useful information for investigating the function of the* OsMsh6* gene in DNA repair and exploiting MMR mutants in induced mutation breeding in rice.

## Supplementary Material

The Supplementary Material contains two parts. One is the mutated bases and their positions originally detected in all materials as listed in sheet 'mutated base', in which the 'R', 'Y','K', 'M' and 'S' represents 'AG', 'CT', 'GT', 'AC' and 'CG', respectively. Another is the data about bases flanking the mutated base as listed in sheet 'flanking base'.

## Figures and Tables

**Table 1:** SLAFs and sequencing data used in this research.

Mutant lines	SLAF numbers	Total depth	Average depth	Total reads	Q30 (%)	GC%
ND6011/G4	10,743	115,810	10.78	180,760	87.88	44.77
ND6011/G5	10,585	91,666	8.66	151,388	88.22	44.76
ND6011/G6	10,572	112,380	10.63	175,846	87.70	45.34
ND6011/G7	10,806	157,011	14.53	250,098	86.68	44.9
NF7784/G4	10,760	124,170	11.54	186,342	87.08	45.15
NF7784/G5	10,685	100,866	9.44	163,096	86.69	44.45
NF7784/G6	10,812	144,665	13.38	242,707	87.14	44.84
NF7784/G7	10,696	114,233	10.68	241,558	86.02	44.85
NF9010/G4	10,767	138,787	12.89	248,652	87.15	44.41
NF9010/G5	10,799	165,225	15.3	249,922	86.71	45.41
NF9010/G6	10,813	172,575	15.96	274,444	87.60	45.08
NF9010/G7	10,798	157,867	14.62	254,224	87.03	44.26
Msh6WT	10,688	177,207	16.58	266,006	88.72	45.27

**Table 2:** Number of mutations in different mutants at different generations.

Mutants	Generation	Mutated bases	Total length (Kb)	Mutation density (1/Kb)
ND6011	G4	15	4297	286.48
	G5	21	4234	201.62
	G6	188	4229	22.49
	G7	219	4322	19.74

NF7784	G4	18	4304	239.11
	G5	30	4274	99.40
	G6	43	4325	144.16
	G7	373	4278	11.47

NF9010	G4	14	4307	307.63
	G5	24	4320	179.98
	G6	36	4325	120.14
	G7	513	4319	8.42

**Table 3:** Number and characteristics of four kinds of mutated bases.

Mutated base	Number of mutations				Type of base substitution		
	Number	Homo.	Hetero.	Homo. & hetero.	Transition	Transversion	Ratio
A	272	27	214	31	198	74	2.68
C	250	29	201	20	194	56	3.46
G	208	16	171	21	166	42	3.95
T	264	14	229	21	195	69	2.80
Total	994	86	815	93	753	241	3.12

**Table 4:** Spectrum of spontaneous mutations.

Base substitution	Events	Frequency (%)
A → G	198	19.92
C → T	194	19.52
G → A	166	16.70
T → C	195	19.62
A → C	41	4.12
C → A	31	3.12
A → T	33	3.32
T → A	30	3.02
C → G	25	2.52
G → C	20	2.01
G → T	22	2.21
T → G	39	3.92

**Table 5:** Ratios of observed/expected frequencies on either side of the mutated base.

Flanking base	−3	−2	−1	Mutated base	+1	+2	+3
A	1.10	1.01	0.85	A	0.93	1.01	0.99
C	0.97	1.13	1.51	A	1.03	1.01	0.78
G	0.81	0.72	0.62	A	0.94	0.75	0.96
T	1.12	1.13	1.01	A	1.10	1.22	1.28
*χ* ^2^_(3)_	4.21	7.71	29.44		1.38	7.59	8.76
*P* (%)	0.240	0.053	1.81 × 10^−6^		0.710	0.055	0.033
A	1.10	0.77	1.20	C	0.98	1.09	1.12
C	1.02	0.88	0.90	C	0.83	0.64	0.96
G	0.80	1.09	0.86	C	1.36	1.25	0.88
T	1.07	1.26	1.04	C	0.83	1.02	1.04
*χ* ^2^_(3)_	3.54	9.10	4.43		11.66	12.46	2.00
*P* (%)	0.316	0.028	0.218		0.009	0.006	0.572
A	0.94	0.88	1.02	G	1.06	1.25	1.19
C	0.94	1.33	1.33	G	0.92	0.77	0.83
G	0.79	0.73	0.71	G	1.02	0.88	0.96
T	1.33	1.06	0.94	G	1.00	1.10	1.02
*χ* ^2^_(3)_	8.23	10.19	10.08		0.50	7.19	3.58
*P* (%)	0.041	0.017	0.018		0.919	0.066	0.311
A	1.12	1.05	1.12	T	1.09	1.15	1.15
C	0.91	0.76	1.14	T	0.61	0.79	0.79
G	0.98	1.12	1.08	T	1.50	1.15	1.15
T	0.98	1.08	0.67	T	0.80	0.91	0.91
*χ* ^2^_(3)_	1.55	5.36	9.91		29.85	6.55	6.55
*P* (%)	0.672	0.147	0.019		1.49 × 10^−6^	0.088	0.088

**Table 6:** Bases with the deviation frequency at least 15%.

Mutated base	Flanking base	Position	Bias%
A	C	−1	51.47
		+3	−22.06
	G	−1	−38.24
		−2	−27.94
		−3	−19.12
		+2	−25.00
	T	+2	22.06
		+3	27.94

T	A	+2	15.15
		+3	15.15
	C	−2	−24.24
		+1	−39.39
		+2	−21.21
		+3	−21.21
	G	+1	50.00
		+2	15.15
		+3	15.15
	T	−1	−33.33
		+1	−19.70

G	A	+2	25.00
		+3	19.23
	C	−1	32.69
		−2	32.69
		+2	−23.08
		+3	−17.31
	G	−1	−28.85
		−2	−26.92
		−3	−21.15
	T	−3	32.69

C	A	−1	20.00
		−2	−23.20
	C	+1	−16.80
		+2	−36.00
	G	−3	−20.00
		+1	36.00
		+2	24.80
	T	−2	26.40
		+1	−16.80
